# Co‐Producing an Intervention Involving Dental Professionals Providing Oral Health Support in a Mental Healthcare Setting

**DOI:** 10.1111/hex.70698

**Published:** 2026-05-28

**Authors:** Masuma Pervin Mishu, Natalia Kika, Emma Tkach, Piyali Sarkar, Anne Krayer, Michelle Horspool, Emily Peckham

**Affiliations:** ^1^ Institute of Epidemiology and Public Health University College London London UK; ^2^ School of Mental Health and Psychological Sciences Kings College London London UK; ^3^ School of Health Sciences Bangor University Bangor UK; ^4^ Sheffield Health Partnership University NHS Foundation Trust Sheffield UK; ^5^ College of Medicine and Health Bangor University Bangor UK

## Abstract

**Background:**

People with severe mental illness (SMI) are nearly three times more likely to lose all their natural teeth and five times more likely to have tooth decay compared to the general population, negatively impacting their physical and mental health and well‐being. There is a need for greater focus on this area in research, policy and practice to reverse this unacceptable inequality. To address this health inequality, we aimed to co‐produce a system‐level intervention to improve oral health in people with SMI.

**Methods:**

Building on our initial work, seven online stakeholder consultations and one in‐person co‐production workshop were conducted to co‐produce a system‐level intervention. Consultations were conducted with a total of 23 stakeholders to discuss different intervention components. The stakeholders included people with lived experience of SMI, their family members and carers, mental and dental healthcare professionals and dental public health consultants. Notes were taken and summarised for each consultation session, and an in‐person workshop plan was drafted based on the online consultations. The final workshop was conducted with 13 participants (10 of whom had participated in the earlier consultation and 3 of whom were new to the study) to determine which intervention components would be feasible and acceptable through discussing the practical requirements and constraints of implementing a system‐level intervention. Detailed notes were taken and analysed using the Acceptability, Practicability, Effectiveness, Affordability, Side‐Effects and Efficacy (APEASE) framework.

**Results:**

Six intervention delivery steps ‐ including suggested intervention components for an integrated model of dental supportwere identified and agreed in the workshop: (1) having dental health professionals visit the mental health setting, (2) initiating conversation around dental health in a mental healthcare setting, (3) providing a brief dental check‐up using a dental mirror, (4) tailoring oral health maintenance‐related advice, (5) providing positive reinforcement and (6) providing continuous engagement and support accessing dental visits. These components were believed to be potentially acceptable to service providers and service users, and feasible to deliver.

**Conclusion:**

We co‐produced a system‐level integrated model of dental support to improve oral health in individuals with SMI. The insights from the co‐production process and resulting recommendations will inform future intervention development.

**Patient and Public Contribution:**

People with lived experience of SMI, their carers/family members, and mental and dental healthcare professionals were involved in every stage of developing the intervention. We conducted a series of consultations with these aforementioned stakeholders prior to the intervention co‐production workshop. Stakeholders provided their perspectives on the intervention components. The stakeholders also joined and contributed to the co‐production workshop and helped design the intervention.

## 1 Introduction

People with severe mental illness (SMI) experience significant oral health inequalities compared to people without SMI, with a high burden of elevated rates of dental caries, tooth loss, and periodontal disease due to multiple barriers [1]. A recent umbrella review found that individuals with SMI are nearly three times more likely to lose all their natural teeth compared to people without SMI [2]. Improving oral health in people with SMI is of paramount importance as poor oral health can have a profound impact on people's general health and quality of life [3], as oral health can affect basic functions like eating and speaking, and social interactions [4].

The causes of oral diseases in people with SMI are likely multifactorial. They may include difficulties with self‐care [5], high dietary intake of sugar, cigarette smoking, tobacco and alcohol consumption [6], and side effects from psychotropic medications (e.g., dry mouth) [7]. People with mental illness are less likely to access dental services for routine dental check‐ups and treatment [5], potentially driven by barriers relating to their mental illness, dental anxiety, past trauma or negative experiences, and the lack of systemic social support in accessing dental care [8]. For example, many people experience difficulty finding a dentist, dealing with long waiting lists, as well as travel and cost being barriers to access.

A systematic review conducted by the research team identified 12 studies published after 2016 that presented interventions for improving oral health amongst people with SMI [9]. This review found that, despite statistically significant changes in plaque scores in some studies following dental education with motivational interviewing and incentives, no clinically meaningful changes in oral health outcomes were reported because of these interventions. A pilot study showed some evidence that the oral health of people with SMI can be improved by a basic oral health intervention carried out by oral hygienists and mental health nurses [10], however, there remains challenges in improving oral health in complex populations through education [9]. Another study tested an intervention on oral health checking within general health checking and found no clear evidence that care coordinators (largely nursing staff) using an oral health checklist improved oral health behaviour or oral health state [11]. An RCT among 111 participants with schizophrenia assessed the effectiveness of an oral health education intervention delivered by dental health professionals in a dental setting and showed statistically significant changes in knowledge and attitude, but no significant improvement in the gingival calculus index (lacking clinical importance). Approximately 80% of the subjects returned to their baseline gingival status by the end of the final follow‐up [12]. None of the studies reported the use of dental services or oral health‐related quality of life. While the capacity of educational oral health interventions appears to be limited, based on the results of these studies, oral health interventions based on educational processes have been found to be important and generally effective methods for improving oral health in other complex populations. For example, Balasooriyan et al. conducted a scoping review of existing interventions to improve oral health among children, and found that interventions with a personalised, educational and culturally sensitive approach, engaging with actors across macro, meso and micro levels, were most effective [13].

There have, however, been advancements in the co‐development of programmes and interventions to improve the oral health of people with SMI. A recent co‐produced Therapeutic Educational Oral‐Health (TEPOH) programme delivered four 90‐min workshops in psychiatric outpatient teams showed good acceptability and fidelity, but at 6 months there were no statistically significant between‐group differences on periodontal scores, decayed and missing teeth scores, oral hygiene scores and patient‐reported outcomes changed minimally [14]. Two recent interventions in this field widen the scope beyond advice‐only education to include peer [15] and link‐worker models [16], offering a more realistic and comprehensive support to improve oral health of people with SMI, which could also be complemented by our proposed intervention.

Given the high prevalence of oral diseases in people living with SMI and the impact of poor oral health on physical and mental health and well‐being, there is a need for a greater focus on this area in research, policy and practice to address and reduce this unacceptable health inequality. There is a paucity of information on the available effective interventions to improve oral health in people with SMI. A previous Cochrane review [17] suggests that people with SMI who received oral health education had statistically better oral hygiene than those who did not receive oral health education. However, the quality of evidence in the small number of trials available was low to moderate. Additionally, previous studies have not addressed the patient, carer and service/healthcare perspective on underpinning personal, interpersonal, and system‐level factor barriers that influence maintaining regular oral care and accessing dental care services in people with SMI. Furthermore, there is a paucity of system‐level interventions addressing these barriers to improve oral health in people with SMI [18]. In this context, we consider how a system‐level intervention can be an effective approach to providing continuous support for people with SMI to maintain good oral health. Such an intervention would be delivered through existing mental healthcare services and would be built on collaboration between mental and dental healthcare services.

Our approach to intervention development, particularly the collaborative approach with dental and mental healthcare professionals, service users with SMI and their carers, is novel in this field of research, adding new and important perspectives about effective educational content and strategies for improving the oral health of people with SMI. Therefore, the aim of this study was to co‐produce a system‐level intervention for people with SMI by collaborating with key stakeholders. Specifically, we outline our methodology in co‐developing an intervention to improve oral health in people with SMI, which is intended to be delivered in a mental healthcare setting.

## 2 Methods

**Figure 1:**
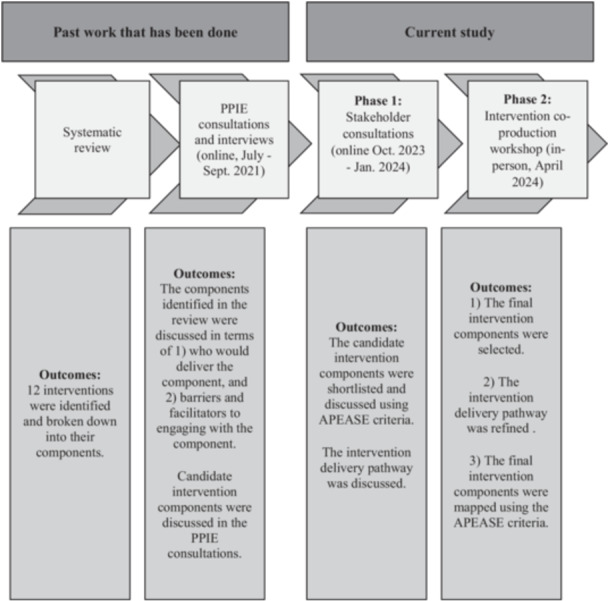
Flow diagram of past and present work. The final intervention components of this study are framed by the Acceptability, Practicability, Effectiveness, Affordability, Side‐Effects and Efficacy (APEASE) criteria.

We adapted the framework developed by Hawkins et al. [19] for co‐producing and prototyping public health interventions. Hawkins' framework outlines three stages to public health intervention production: (1) evidence review and stakeholder consultation, (2) co‐production and (3) prototyping. Our previous work, together with the current work reported in this paper, completes the first two stages of Hawkins et al.'s framework as shown in Figure 1. This work included selecting the candidate intervention components (Appendix S1: Figure 1) and creating a topic guide via the patient public contribution. We used the topic guide to ask about candidate intervention components in detail for final selection.

### 2.1 Previous Work

We conducted a systematic review of all available oral health interventions, followed by 11 stakeholder consultations and 17 dyadic and one‐to‐one interviews between July and September 2021 to explore their views on these existing interventions. The methods and other findings of the interviews were published elsewhere [18, 20]. We identified that integrating dental support into mental healthcare services (e.g., community mental healthcare and early intervention of psychosis) could be invaluable, especially considering the complexity of the barriers this population faces in maintaining oral hygiene and accessing dental services. Several initial candidate intervention components were identified in the consultations (Appendix S1: Figure 1).

To progress and finalise the initial candidate intervention components and critically develop a pathway for delivering the system‐level dental health intervention, we conducted additional consultations with stakeholders, feeding into a co‐production workshop, all of which we describe in this current paper. As shown in Figure 1, the findings reported here include further stakeholder consultations for shortlisting the candidate intervention components (Phase 1) and the co‐production of an intervention through an in‐person workshop (Phase 2).

### 2.2 Current Work

#### 2.2.1 Phase 1: Stakeholder Consultations

Building on our previous work, seven online stakeholder consultations (*N* = 23) were conducted to shortlist different intervention components. In the consultations, we explored stakeholders' views on the previously identified candidate intervention components, which are listed in Appendix S1: Figure 1. We asked open questions such as ‘What are your thoughts on the potential effectiveness of this intervention component?’ In these consultations, we asked stakeholders to share their thoughts on the candidate intervention components and proposed intervention delivery pathways, which were informed by initial consultations conducted in 2021, as well as their views on the acceptability and feasibility of the proposed intervention delivery pathways. It was particularly important to us to hear the perspectives of service users during these consultations, as their views can be difficult to attain yet are highly valuable to assess the acceptability of an intervention [18]. Thus, participant stakeholders included (1) mental health service users with lived experience of SMI, (2) family members and carers of service users with SMI, (3) professionals from mental and dental health backgrounds, including psychiatrists, mental health nurses, dental therapists, dentists and dental public health professionals, and (4) dental public health consultants. Representation of views of mental health service users with lived experience of SMI and their carers is particularly important; however, they are hard‐to‐reach groups, and we attempted to ensure diversity and representation of these groups. Stakeholders were recruited through the research team's professional networks by sending out emails to their health professional contacts, and then recruiting additional stakeholders (other health professionals, people with SMI and family members and carers of people with SMI) through a snowballing strategy, which is generally used to contact hard‐to‐reach populations [21]. In total, we had three mental health service users join these stakeholder consultations (two from ethnic minority groups), and we had two carers join (one from an ethnic minority group). Commissioners were not recruited for these consultations due to a lack of time and resources, and because the aim of this phase of the study was not to explore the cost‐effectiveness or commissioning or implementation pathways of the proposed intervention, but rather to co‐produce an intervention. Our intention was to explore the perceived feasibility of delivering this intervention with practitioners; future work will involve commissioners as important stakeholders in understanding the implementation and commissioning pathways of this intervention.

Online consultations lasted 60 min. All the participants were offered a gift voucher as a token of thanks for taking part in the consultations. Detailed notes were taken during each online consultation by MPM and EP. Notes were organised by a stakeholder group. The organised notes were then used, alongside the Acceptability, Practicability, Effectiveness, Affordability, Side‐Effects and Efficacy (APEASE) criteria to develop the structure for the topic guide that was used in the following in‐person co‐production workshop. APEASE is a framework used to assess whether an intervention is feasible, appropriate, and likely to be suitable and successful for its purpose [22]. The topic guide—as well as the analysis framework for notes taken during the subsequent co‐production workshop—used an adapted version of the APEASE framework, relying on the first four criteria of APEASE: Acceptability, practicability, effectiveness and affordability. Side‐effects and equity were not examined because the study focused on the co‐production and early development of an intervention rather than its implementation or outcome evaluation. At this formative stage, the aim was to explore the feasibility and perceived suitability of the proposed intervention components. The principle of equity and its importance are one of the cornerstones of our work [23].

#### 2.2.2 Phase 2: Co‐Production Workshop

Following the online consultations, an in‐person co‐production workshop was conducted in line with Hawkins et al.'s framework [19]. The workshop included key stakeholders identified in Stage 1 consultations (*n* = 10) plus three additional ones.

The full‐day in‐person event was facilitated by two members (MPM and EP) of the study team. The purpose was to agree on a final list of candidate intervention components and the intervention pathway that would be perceived as both feasible and acceptable to stakeholders. Discussions on feasibility and acceptability were based on the four APEASE criteria outlined above.

Detailed notes were taken during the full‐day workshop. Ethical approval was obtained from the UCL Research Ethics Committee for the workshop (Phase 2). As the consultations in Phase 1 of the co‐development process fall under public and patient involvement (PPI) in the design of research and do not involve collecting personal data, ethical approval was not required, as per UCL's Research Ethics guidelines. We followed the UK Public Involvement Standards throughout [24]. All participants were offered a gift voucher as a token of thanks for participating in the workshop, along with reimbursement of their travel expenses.

### 2.3 Data Analysis

The notes from the co‐production workshop were analysed using Ritchie et al.'s approach to framework analysis for intervention development [25]. This analysis approach follows six steps: (1) familiarisation, (2) coding, (3) development of an analytic framework, (4) applying the framework, (5) charting the data and (6) interpreting the data.

1. Familiarisation of the workshop data involved grouping the data into two initial semantic themes: (1) barriers to intervention development and delivery, and (2) facilitators to intervention development and delivery. Data familiarisation also included organising the notes by each participant, keeping in mind the different stakeholder groups of each participant.
2. Data were then coded based on a priori themes taken from the adapted APEASE framework, using the first four criteria: Acceptability, Practicability, Effectiveness and Affordability. The semantic themes of barriers and facilitators to intervention development and delivery for each participant.
3. We developed our framework for this data analysis from the APEASE framework, using the first four criteria: Acceptability, Practicability, Effectiveness and Affordability. The adapted APEASE framework was applied to the note data, now semantically coded as either a facilitator or barrier for intervention development and delivery. Data were analysed on an individual participant basis at this stage.
4. The data were then charted based on each of the adapted APEASE criteria, and organised under each APEASE criterion by the three main stakeholder groups: dental health professionals, mental health professionals and people with SMI.
5. The analysed data, now charted by APEASE criteria and stakeholder group, led to the final selection of the candidate intervention components (Figure 1).

## 3 Results

### 3.1 Stakeholder Consultations

Twenty‐three stakeholders (*N* = 23) took part in the stakeholder consultations. Please see Appendix S1: Table 1a for a stakeholder and demographic breakdown of the stakeholder participants. We had originally planned to hold a separate consultation for each group; however, due to a higher level of interest than expected and logistical challenges, groups were mixed. We found that the mixing of stakeholder groups allowed for greater depth and exploration of the topics discussed in the stakeholder consultations, as well as stakeholder satisfaction, highlighting the acceptability and feasibility of this kind of stakeholder consultation.

### 3.2 Co‐Production Workshop

Thirteen participants (*N* = 13) took part (10 participants had previously taken part in the stakeholder consultations), including people with SMI (*n* = 1), family member and carer of people with SMI (*n* = 1), dental health professionals (*n* = 5, including dental therapists, dentists and dental public health professional), mental health professionals (*n* = 2, including a mental health nurse and psychologist) and a public health consultant (*n* = 1). Public health researchers from our team (*n* = 3) were considered as part of this co‐production workshop. Please see Appendix S1: Table 1b for the demographics of the workshop participants.

**Figure 2:**
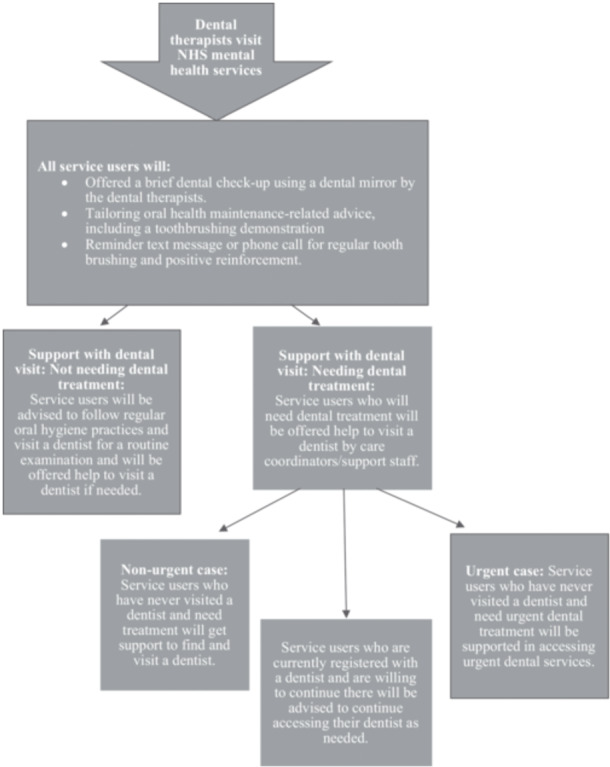
Proposed intervention delivery pathway.

The results of the co‐production workshop led to the agreement of six finalised intervention components (Table 1) and proposed intervention delivery pathway (Figure 2).

**Table 1:** Finalised intervention components with explanation.

Finalised intervention component	Explanation
Dental health professionals visiting a mental healthcare setting.	It was agreed that integrating dental support delivered by dental healthcare professionals in a mental healthcare setting would be helpful, considering the complexity of the barriers people with SMI face in maintaining oral health and accessing dental services. A dental therapist could make the visit as this might help overcome some of the barriers.
Initiation of conversation around dental health in a mental healthcare setting.	When people with SMI come to mental healthcare services, it is suggested that a member of the community mental health team could initiate a conversation about oral health and briefly mention the importance of oral health.
	This would cover:
	How they perceive their oral health, How satisfied they are with their oral health, When they last visited a dentist.
	The member of the community mental health team should inform the service user that there is a dental therapist visiting the mental health clinic, whom they could speak to about their oral health if they wish. If this offer of dental support were accepted, they would be introduced to the dental therapist.
	As part of the conversation, dental therapists will have tailored discussions about the person's oral health, oral hygiene practice, and dietary habits regarding sugary food and drink intake.
Brief dental check‐up using a dental mirror.	The dental therapists should offer to do a brief dental check‐up using a dental mirror. If the service user agrees, then the dental check‐up should be conducted. If consent is not provided, the remaining components of the intervention will still be carried out as planned.
Tailored oral health maintenance‐related advice.	The dental therapists should provide tailored oral health maintenance‐related advice, which will include:
	Importance of regular tooth brushing. Tooth brushing instruction with details on when and how to brush, along with a practical demonstration tailored to their specific needs. Explain the role of dietary sugar and its relation to oral health, and give related tailored advice regarding reducing the frequency and amount of sugary food and drink consumption to maintain good oral health and reduce tooth decay/dental caries. Explain the role of tobacco and alcohol and their relation to oral health, and give tailored advice where needed. Because certain anti‐ psychotic medications can cause a dry mouth, therapists should make the service users aware of this issue and recommend suitable solutions. Explain how the side effects of some antipsychotic medications are related to oral health, such as dry mouth, and how to deal with them. Explain the importance of routine dental check‐ups for good oral health.
Reminder text message or phone call, on regular tooth brushing and positive reinforcement	For the next 4 weeks, the service users should receive a weekly phone/text message (as they prefer) with positive reinforcement on regular tooth brushing.
	Their family members will be involved in the intervention, if the service users wish, to encourage and remind them to maintain regular oral hygiene and accompany them to visit a dentist.
Support with dental visits	Oral healthcare should be included in their care plan. Members of the community mental health team, with support from the dental therapists, should help facilitate access to a dentist or community dental services.
	Those having regular dental visits will be encouraged to maintain routine dental checkups and seek dental treatment (if needed). Those who never visited a dentist and do not need urgent treatment will be supported to find a dentist for doing and maintaining routine dental checkups and seek dental treatment (if needed). Those who need urgent dental treatment will be assisted to seek emergency dental care.

The finalised intervention components in Table 1 were analysed using the adapted APEASE criteria: Acceptability, Practicability, Effectiveness and Affordability. Table 2 breaks down each finalised intervention component by APEASE criteria and participant group, highlighting the semantic themes we identified during our analysis: barriers and facilitators to the development and delivery of this intervention.

**Table 2:** Mapping of the barriers and facilitators for each intervention delivery step, based on the adapted APEASE criteria: Acceptability, Practicability, Effectiveness and Affordability.

Finalised intervention component	APEASE criteria			
	Acceptability	Practicality	Effectiveness	Affordability
Dental healthcare professionals (DHP) visiting the mental healthcare (MHC) setting	**DHP:** Acceptable to visit the mental healthcare setting to deliver the intervention. **MHP:** Very welcoming to have dental therapists come to MHC care settings to deliver the intervention as they are used to working in multi‐disciplinary teams. **People with SMI:** Getting some dental care support in mental health services would be welcome	*Concerns from both **DHP's** and **MHP's**: (1) the low availability of dental therapists and mental health service care coordinators, (2) intervention requires a suitable location (secondary mental healthcare setting)*. **MHP:** Raised several points about the practicality and acceptability of this intervention component: (1) expressed the importance of giving service users adequate notice of the DHP visiting, and (2) appropriate and private spaces in the mental health setting should be available due to the sensitivity of dental check‐ups. **People with SMI:** The commute to dental clinics can be challenging, so it would be beneficial to get advice on dental care in MH setting.	**DHP:** Should be trained appropriately on softer communication skills and trauma‐informed care before delivering the intervention to the service users in MHC. **MHP:** Should be trained appropriately and need to work in close collaboration with DHP to make the intervention effective **People with SMI:** Having dental check‐ups in an MH setting could make patients more comfortable.	**DHP:** Discussion needed with commissioners regarding the availability of funding for conducting clinical dental check‐ups in mental health settings by dental therapists. Approach should be effective for gaining funding from commissioners if targeted to the high needs group of people for preventive dental measures. *Both **DHP's** and **MHP's** brought up the question of funding and commuting for dental therapists, as these may pose barriers to collaboration between DHP's and MHP's*. **People with SMI:** NR*
Initiation of conversation around dental health in mental healthcare settings by mental healthcare professionals (MHP)	**DHP:** Acceptable **MHP:** Acceptable **People with SMI:** No one is currently asking service users about their oral health in MHC, so they would be happy to be asked	**DHP:** Rather than using a structured questionnaire or checklist, these topics could be explored through an open conversation aimed at understanding the service user's dental health needs, their self‐perceived oral health status, level of satisfaction with their oral health, and patterns of dental service use. **MHP:** First point of contact, so could ask initial questions, following which they would speak to the service user about seeing a dental therapist who could then provide more tailored advice. **People with SMI:** These questions would be welcome.	*Both **DHP's** and **MHP's** suggested incorporating questions around oral health into the physical health assessment, which is routinely conducted in mental health services*. **People with SMI:** Emphasised the importance of the questions being asked in a sensitive manner.	**DHP:** NR **MHP:** NR **People with SMI:** NR
Clinical dental check‐up using a dental mirror	**DHP:** Conducting a dental check‐up with a dental mirror only without a dental chair will be acceptable. **MHP:** Emphasised the need to consider barriers to attending check‐ups experienced by the service users, such as previous negative experiences with the dentist and dental anxiety. **People with SMI:** Highlighted that many may feel vulnerable due to the check‐up, so this should be approached with sensitivity by the DHP in a trauma‐informed manner.	**DHP:** Feasible **MHP:** Feasible **People with SMI:** should be approached with sensitivity. However, on subsequent dental check‐ ups, they may feel more comfortable and build trust with the team.	**DHP:** Brief check‐up could help deliver tailored oral hygiene advice and identify the need for further dental visits and DHP could then inform the service user about how to see the dentist. **MHP:** If needed, mental healthcare coordinators can help with visiting a dentist. **People with SMI:** NR	**DHP:** Commissioning route needs to be explored. **MHP:** NR **People with SMI:** NR
Tailored oral health maintenance‐related advice	**DHP:** Tailored advice on oral hygiene by DHP after the dental check‐up will be important. **MHP:** Acceptable **People with SMI:** The advice will be welcome if delivered in a non‐judgmental and non‐authoritative manner.	**DHP:** Could provide a toothbrushing demonstration ‐ format of demonstration can vary (video, leaflet, model) **MHP:** It must also be ensured that all information is provided in small, manageable sections rather than all at once to avoid information overload and confusion. **People with SMI:** Providing manageable sections of information is acceptable to those experiencing mental health crises.	**DHP:** Continuous oral health support by offering gentle reminders for follow‐up every 3‐6 months. **MHP:** Dental advice should be shared with carers so they can encourage and support the service users at home. **People with SMI and carer:** Advice should also be provided to family members or carers (if requested by the service users) to help reminding them.	**DHP:** To increase cost‐effectiveness and practicality, dietary sugar and tobacco‐alcohol advice relating to oral health should be embedded into routine advice as part of the physical health check‐up, with signposting as needed. **MHP:** NR **People with SMI:** Can be provided with fluoridated toothpaste after their clinical dental check‐up to boost their motivation as some groups of the population may find it unaffordable and inaccessible.
Reminder text message or phone call, and positive reinforcement	**DHP:** Helpful to develop oral hygiene habits, but support is needed from MHP through reminders via phone call or text **MHP:** Expressed the importance of asking individual service users whether they would like to receive a text message or call reminder, and keeping this aspect more tailored to individual preferences. **People with SMI:** should be tailored to individual preferences	**DHP:** External help for administrative tasks will be helpful (e.g. from peer support workers/care coordinators) **MHP:** Care coordinators in mental health services could offer follow‐up phone calls/ sending text message. **People with SMI:** Family members could be included if the service users prefer	**DHP:** Effective for establishing habits. **MHP:** Effective for establishing habits. **People with SMI:** Effective for establishing habits.	**DHP:** NR **MHP:** NR **Service users:** NR
Support with dental visits	**DHP:** Acceptable, but the specific details of the referral pathways still need to be explored. **MHP:** Reviewing history of dental visits should be part of routine physical health check. **People with SMI:** Any support with a dental visit will be welcomed	**DHP:** Dental check‐ups at community dental services could be a barrier due to long waiting lists. **MHP:** Suggested that it would be feasible to include oral health in the mental healthcare plan. Doing so could enable care coordinators to provide additional support for service users in accessing dental services. **People with SMI:** Getting support for dental visits for routine check‐ups or dental treatment will be beneficial.	**DHP:** Robust collaboration and an effective communication channel between mental and dental care services are crucial for effectiveness. **MHP:** Service users should be aware of the benefits of routine dental visits and the exemption of charges for patients. **People with SMI:** Need for publicity to circulate messages on available benefits to service users with mental illness for free dental treatments.	**DHP:** Dental check‐ups are costly. Dental therapists, hygienists, and dental nurses could be trained for the initial dental checkup of service users with mental illness in a Community Dental setting rather than having a dentist conduct the check‐up. **MHP:** Collaboration amongst commissioners for funding is needed in case of inter‐departmental activities, so that the DHP and MHP can work collaboratively to support service users to visit a dentist. **People with SMI:** Support with paperwork for getting free dental treatment would be beneficial.

*Note:* DHP refers to dental health professionals, and MHP refers to mental health professionals in this table.

* NR: no response.

The finalised intervention components were generally acceptable and feasible across stakeholder groups (Table 2). Some of the Intervention components were not selected due to service users' and providers' feedback. For example, service users' completion of a dental checklist was not selected due to their feedback that they would prefer a conversation‐based mode of communication rather than a tick‐box exercise, and simply completing a dental checklist without further support is unlikely to improve their oral health. Dental professionals considered that better information on the oral health condition of service users could be obtained through a brief oral examination by dental therapists. Intervention components, such as service users repeating the brushing demonstration, and service users practising tooth‐brushing in front of the dental team, were not selected as the service users considered that repeating the brushing demonstration, and service users practising brushing in front of the dental team, were too childish, and they would not feel comfortable doing so in this setting.

The main barriers discussed were the capacity of mental and dental healthcare staff, the process of initiating conversations around dental health into routine check‐ups in mental health services, using softer and non‐patronising communication while providing the advice, and approaching dental check‐ups in a sensitive and trauma‐informed manner. Soft communication and non‐patronising approaches when providing dental advice were particularly vital to the acceptability of the intervention among service users. Though this is not explicitly shown in Figure 2, we considered providing training to the mental and dental healthcare professionals to deliver the intervention using softer communication skills in the proposed intervention delivery pathway as recommended by the service users. The mental and dental healthcare professionals reported a lack of training opportunities in this area, which we will address by providing the training.

Overall, all stakeholder groups found that the intervention would have the potential to improve oral health in people with SMI.

The overall result of the stakeholder consultations and the co‐production workshop was the development of an oral health intervention for mental health settings. An animated video that explains the intervention in more detail can be accessed at the link in Appendix S2. An intervention booklet can be found in Appendix S3. However, as we are taking a flexible approach to co‐producing the intervention, and based on this proposed intervention, we will move to the next phase of prototyping. Discussions around the frequency or staging of dental check‐up/advice provision/support to access dental care will be addressed in the prototyping phase of future intervention co‐development.

## 4 Discussion

Following a co‐production process with multiple stakeholders, we identified a potential system‐level intervention to improve oral health of mental health service users with lived experience of SMI, whereby dental health professionals provide dental support in a mental healthcare setting. Within this system‐level intervention, we identified six finalised intervention components that can be delivered through this intervention: (1) having dental health professionals visit the mental health setting, (2) initiating a conversation around dental health by mental health professionals in a mental healthcare setting, (3) providing a brief dental check‐up using a dental mirror by dental health professionals, (4) Giving tailored oral health maintenance‐related advice, (5) Reminder text message or phone call and positive reinforcement and (6) providing continuous support in maintaining healthy oral health and support with dental visits.

While we have defined these as separate steps, it is possible that there will be some fluidity between the steps. We acknowledge that these intervention components present an ideal intervention delivery process, but that may not be possible in practice due to the very real challenges in accessing dental care and the variable services across the country [26]. Furthermore, we recognise that our proposed intervention delivery requires the resources of existing services, which would ultimately spread already limited services more thinly. The benefits, costs, feasibility and acceptability of this intervention must be explored in more depth in subsequent studies.

Whilst some of the steps have been explored in other studies, to our knowledge, this is the first intervention that combines all these steps into one single intervention. For example, ‘The Three Shires’ study [11] explored the use of an oral health checklist in routine practice for people in early intervention for psychosis. This alone was found not to be effective, but we do not know whether a checklist is an effective component of a multi‐component intervention. Our proposed intervention would benefit from further patient interviews, and we propose to do this within a feasibility study, where we will interview people who have received the intervention. Cost‐effectiveness would be further addressed in an RCT.

In addition, while Palmier‐Claus et al. [16] offered participants in their study an oral health examination with a dental therapist, this was offered as one of the outcome measures of the trial rather than as a component of an intervention, as we are proposing. Therefore, we do not know whether an oral health examination would be effective when delivered as part of a multi‐component intervention. Finally, DHPs are increasingly being trained in mental health, and dental therapists who work in community dental services are trauma informed [27]. This overlap in training and service provision is an important shift in this field, and as part of the training to deliver our proposed intervention, we would build upon this transition and train dental therapists in mental health.

A study by Agarwal et al. in India examined dental services embedded within a mental healthcare setting, including the engagement of family members in supporting mental health service users [12]. While there is no such embedded dental service within a mental healthcare setting in the United Kingdom, it may be a systemic shift in healthcare in the coming years. The service users in our study considered that more practical support in finding and accessing a dentist/dental service would be helpful.

Regarding intervention component 2 (initiating conversation around oral health in a mental health care setting), whilst NHS England suggests that oral health is included in physical health checks for people with SMI [26], this is not a mandatory item; in addition, physical health checks are generally delivered in primary rather than secondary care. In terms of intervention component 4 (tailored oral health maintenance‐related advice), it is possible that oral health advice may be provided via digital platforms, but we do not know at this time whether people with SMI are likely to engage with advice provided by digital platforms. However, we would underscore the importance that advice via digital platforms be tailored to the person's individual needs. We further acknowledge that people with SMI often lack digital skills [28], which means that they may not have the skills required to access digital interventions. Yet, the potential of digital interventions to reach complex populations such as people with SMI is still valuable and important to explore.

Our proposed intervention aligns with the strategy suggested by *The Right To Smile* [23], a consensus statement to tackle oral health inequalities in people with SMI, which stated ‘the right to have their oral health valued and supported; the right to consider oral health from the start of their mental health difficulties and treatment; the right to receive information and advice on issues relating to their oral health to make informed decisions; and the right to regular dental check‐ups and dental treatment if they need it’. The translation of this strategy into practice is complex, but the proposed intervention and learnings from co‐production processes may provide a blueprint. For example, a general finding that cuts across all intervention components is that there is a need for appropriate training for both mental and dental care providers to deliver the intervention, which aligns with the right for empowering a person to consider oral health from the start of their mental health difficulties and treatment. Furthermore, we found that it is highly important that dental and mental health professionals delivering the intervention are trained in trauma‐informed care, which further aligns with this sentiment.

As the evidence showed that an educational intervention aimed at the individual failed to achieve significant long‐term clinical oral health improvement [9], a dental and mental healthcare system‐level support system/intervention was recommended in our stakeholder consultations. This recommendation aligned with recent studies developing oral‐health educational programmes and interventions for people with SMI [14, 16].

### 4.1 Strengths and Limitations

The current study provides important insights into how a system‐level intervention to improve oral health in people with SMI could be co‐produced that could be delivered in a mental healthcare setting. We incorporated a wide range of stakeholders to get their perspectives. However, the study has some limitations. We had limited stakeholder representation from different ethnic backgrounds and non‐English‐speaking groups. While two service users and one carer were from ethnic minority groups, they could speak English. Funding limitations for translators meant that we could not include non‐English speakers. Due to resource constraints, we were unable to involve commissioners in our stakeholder consultation at this stage. Future work, looking at prototyping and implementation, will focus on this. The co‐production workshop would likely have benefited from more engaging presentations from service users and mental health professionals.

Our future study will develop a blueprint for the intervention and related training materials. Intervention evaluation and monitoring will also need to be developed going forward. There were discussions about evaluating intervention outcomes assessed by DHPs (dental plaque measurements, bleeding on probing, periodontal pocket depth), but this type of evaluation was deemed infeasible given that this intervention is intended for delivery in the mental healthcare setting. Evaluating and monitoring intervention outcomes using MHP's was not discussed in depth and should be covered in future research. We intend to apply for further funding to prototype the intervention and conduct a full feasibility and acceptability trial of this intervention.

### 4.2 Practice and Policy Recommendations

This proposed study will lead to the development of a blueprint for an intervention to improve oral health among people with SMI, ultimately helping reduce the health inequities faced by this population. Due to COVID‐19, there is a greater chance of increasing the dental health gap due to a lack of access to dental care services. Therefore, there is an urgent need to address this challenge and start working on developing a tangible and effective intervention that could be offered by the NHS to improve oral health for this population, which will be acceptable to service users and providers. The potential commissioning route and the related economic model of delivering the intervention need to be investigated. Therefore, future research should test the feasibility and effectiveness, cost‐effectiveness, and implementation of the intervention.

## 5 Conclusion

Poor oral health has a negative impact on the physical and mental well‐being of people with SMI. There is a need for greater focus on this area in research, policy and practice to reverse this unacceptable but often neglected health inequality. We co‐produced a system‐level intervention to improve oral health in people with SMI. In the next stage, we will finalise the blueprint of the intervention and develop related training materials and aim to test the feasibility, effectiveness, cost‐effectiveness and implementation of the intervention.

## Author Contributions

**Masuma Pervin Mishu:** project administration, investigation, funding acquisition, conceptualisation, methodology, writing – original draft, writing – review and editing. **Natalia Kika:** formal analysis, writing – original draft, methodology, data curation. **Emma Tkach:** writing – review and editing, visualisation, methodology, validation. **Piyali Sarkar:** formal analysis, methodology, writing – review and editing. **Anne Krayer:** writing – review and editing, formal analysis, data curation, methodology. **Michelle Horspool:** conceptualisation, writing – original draft, writing – review and editing, methodology, validation. **Emily Peckham:** conceptualisation, investigation, writing – original draft, methodology, writing – review and editing, supervision.

## Ethics Statement

Ethical approval was obtained from the University College London Research Ethics Committee (ref no. 27717/001).

## Consent

Informed consent was gained from all participants in this study.

## Conflicts of Interest

The authors declare no conflicts of interest.

## Supporting information

Supporting File 1

Supporting File 2

Supporting File 3

## Data Availability

The data that support the findings of this study are available on request from the corresponding author. The data are not publicly available due to privacy or ethical restrictions.
