# Supplementary material for: Co‐Producing an Intervention Involving Dental Professionals Providing Oral Health Support in a Mental Healthcare Setting
Source: Health Expect. 2026 May 28;29(3):e70698. doi: 10.1111/hex.70698 (PMC13239521; doi:10.1111/hex.70698)
Supplement: Supplementary file 1 — Supporting File 1 [file HEX-29-e70698-s001.docx]

# Appendix I:

**Figure 1:** List of candidate intervention components.

**List of identified intervention components via systematic review**

1. Ask about oral health in mental health care services.

2. Completion of a dental check-list.

3. Clinical dental check-up by dentist/dental therapist who will visit the mental health care.

4. Tailored dental education on maintaining oral hygiene/regular tooth brushing.

5. Instruction on general tooth brushing technique.

6. Tailored instruction and advice using a mirror.

7. Brushing demonstration on dental model.

8. Service users repeat the brushing demonstration.

9. Service users practice brushing in front of the dental.

10. Show video on brushing technique.

11. Advice on reducing sugary food and promote healthy eating.

12. General advice on tobacco cessation and sensible alcohol drinking.

13. Discuss dry mouth as a side effect of medication, and how to reduce its effect.

14. Different types of reminder systems for brushing reminders

15. Positive reinforcement: mental health professionals support with positive reinforcement and reminder of brushing (weekly phone call or text for the next four weeks).

16. Encourage visiting a dentist for routine check-up and early dental treatment if needed and help support of finding a dentist and visiting a dentist.

17. Engage family members and carers if the service users want.

1. Ask about oral health in mental health care services.

2. Clinical dental check-up by dentist/dental therapist who will visit the mental health care.

3. Tailored dental education on maintaining oral hygiene/regular tooth brushing.

4. Instruction on general tooth brushing technique.

5. Tailored instruction and advice using a mirror.

6. Brushing demonstration in dental model.

7. Show video on brushing technique.

8. Advice on reducing sugary food and promote healthy eating.

9. General advice on tobacco cessation and sensible alcohol drinking.

10. Discuss dry mouth as a side effect of medication, and how to reduce its effect.

11. Positive reinforcement: mental health professionals support with positive reinforcement and reminder of brushing (weekly phone call or text for the next four weeks).

12. Encourage visiting a dentist for routine check up and early dental treatment if needed and help support of finding a dentist and visiting a dentist.

13. Engage family members and carers if the service users want.

**Recommended intervention components selected by the PPIE consultations**

**Six main intervention delivery steps with potential intervention components discussed in the in-person co-design workshop**

**1. Dental health professionals visiting a mental health setting**

**2. Initiation of conversation around dental health in mental health care setting**

**3. Brief dental check-up using a dental mirror**

**4. Tailored oral health maintenance-related advice including a tooth brushing demonstration**

**5. Reminder text message or phone call on regular tooth brushing and positive reinforcement**

**6. Support with dental visits**

# **Table 1a:** Description of the stakeholder consultation (phase 1 of the current study) participants.

| **Date of stakeholder consultation** | **Background of the stakeholders (N=23)** | **Gender** |
| --- | --- | --- |
| 18.10.2023 | People with SMI (n=1)  Mental health professional (n=2) | Female (n=2)  Male (n=1) |
| 28.11.2023 | People with SMI (n=1)  Family member/carer of people with SMI (n=1)  Mental health professional (n=1) | Female (n=2)  Male (n=1) |
| 1.12.2023 | Mental health professionals (n=4) | Female (n=2)  Male (n=2) |
| 12.12.2023 | People with SMI (n=1)  Dental health professionals (n=3) | Female (n=2)  Male (n=2) |
| 15.12.2023 | Dental health professionals (n=4) | Female (n=3)  Male (n=1) |
| 19.12.2023 | Family member/carer of people with SMI (n=1)  Mental health professional (n=1) | Female (n=2) |
| 18.1.2024 | Dental health professionals (n=3) | Female (n=3) |

**Table 1b:** Description of co-production workshop (phase 2 of the current study) participants. The co-production workshop was conducted on 04.24.2024 in person.

| **Background of the co-production workshop participant (N=13)** | **Gender** |
| --- | --- |
| People with SMI (n=1)  Family member and carer of people with SMI (n=1)  Mental health professionals* (n=2)  Dental health professionals** (n=5)  Dental Public Health Consultant (n=1)  Public health researchers (n=3)  *One of the mental health professionals was new to the group of stakeholders and had not been part of the stakeholder consultations.  **Dental health professionals were new to the group of stakeholders and had not been part of the stakeholder consultations | Female (n=10)  Male (n=3) |
