# Supplementary material for: Co‐Producing an Intervention Involving Dental Professionals Providing Oral Health Support in a Mental Healthcare Setting
Source: Health Expect. 2026 May 28;29(3):e70698. doi: 10.1111/hex.70698 (PMC13239521; doi:10.1111/hex.70698)
Supplement: Supplementary file 2 — Supporting File 2 [file HEX-29-e70698-s003.docx]

# Appendix II:

Animated video explaining the intervention: <https://www.youtube.com/watch?v=hYy6mUmer9A>
