# Supplementary material for: Co‐Producing an Intervention Involving Dental Professionals Providing Oral Health Support in a Mental Healthcare Setting
Source: Health Expect. 2026 May 28;29(3):e70698. doi: 10.1111/hex.70698 (PMC13239521; doi:10.1111/hex.70698)
Supplement: Supplementary file 3 — Supporting File 3 [file HEX-29-e70698-s002.pdf]

Enabling Service Users with Severe Mental Illness to Learn about and Engage (SMILE) with Good Oral Health

An Intervention to improve oral health in people with severe mental illness

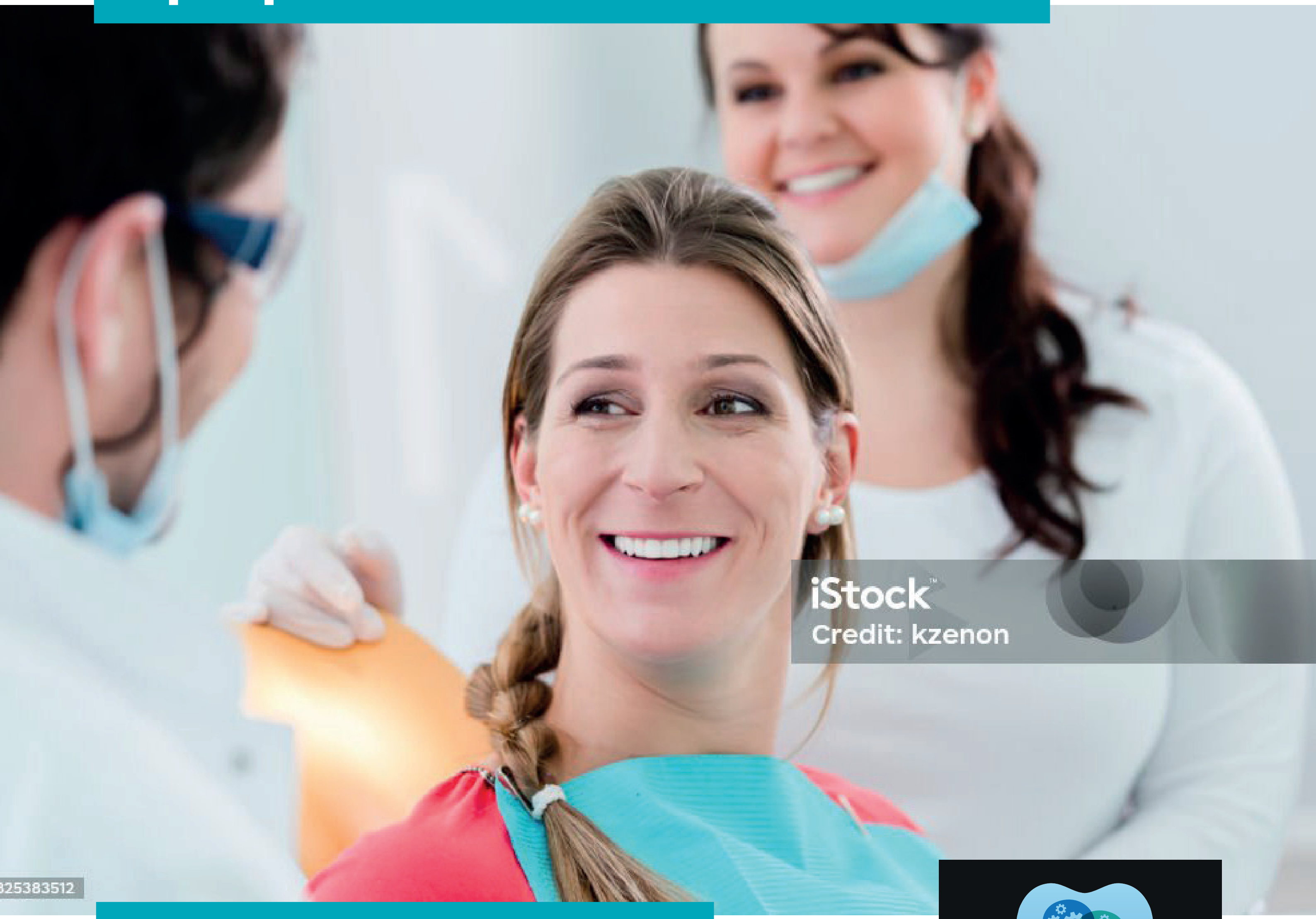

Providing support from dental professionals in a mental health care setting

SMILE with Good Oral Health

People with Severe Mental Illness to Learn about and Engage with Good Oral Health

Introduction

Oral health is integral part of general health.

Poor oral health has negative impact on physical and mental health and well-being. Oral health can affect basic functions like eating and speaking, and social interactions. It can negatively affect individuals' self-esteem, and functioning. Poor oral health is also linked with systemic conditions like cardiovascular disease and diabetes.

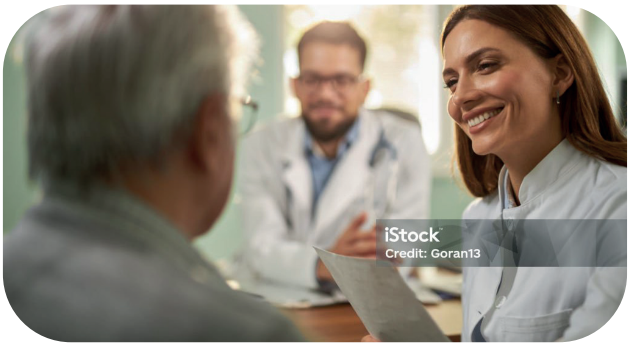

People with severe mental illness (SMI) have poorer oral health compared to people without severe mental illness due to multiple barriers related to maintaining regular oral hygiene and access to dental services. Oral health has largely been overlooked, despite higher rates of dental caries, periodontitis and tooth loss. There is a need for greater focus on this area in research, policy and practice to reverse this unacceptable but often neglected health inequality. To reduce this huge oral health inequality and to help improve oral health in people with severe mental illness, we are co-developing a system level intervention with dental and mental health care professionals, public health experts and people with lived experience and their family members and carers.

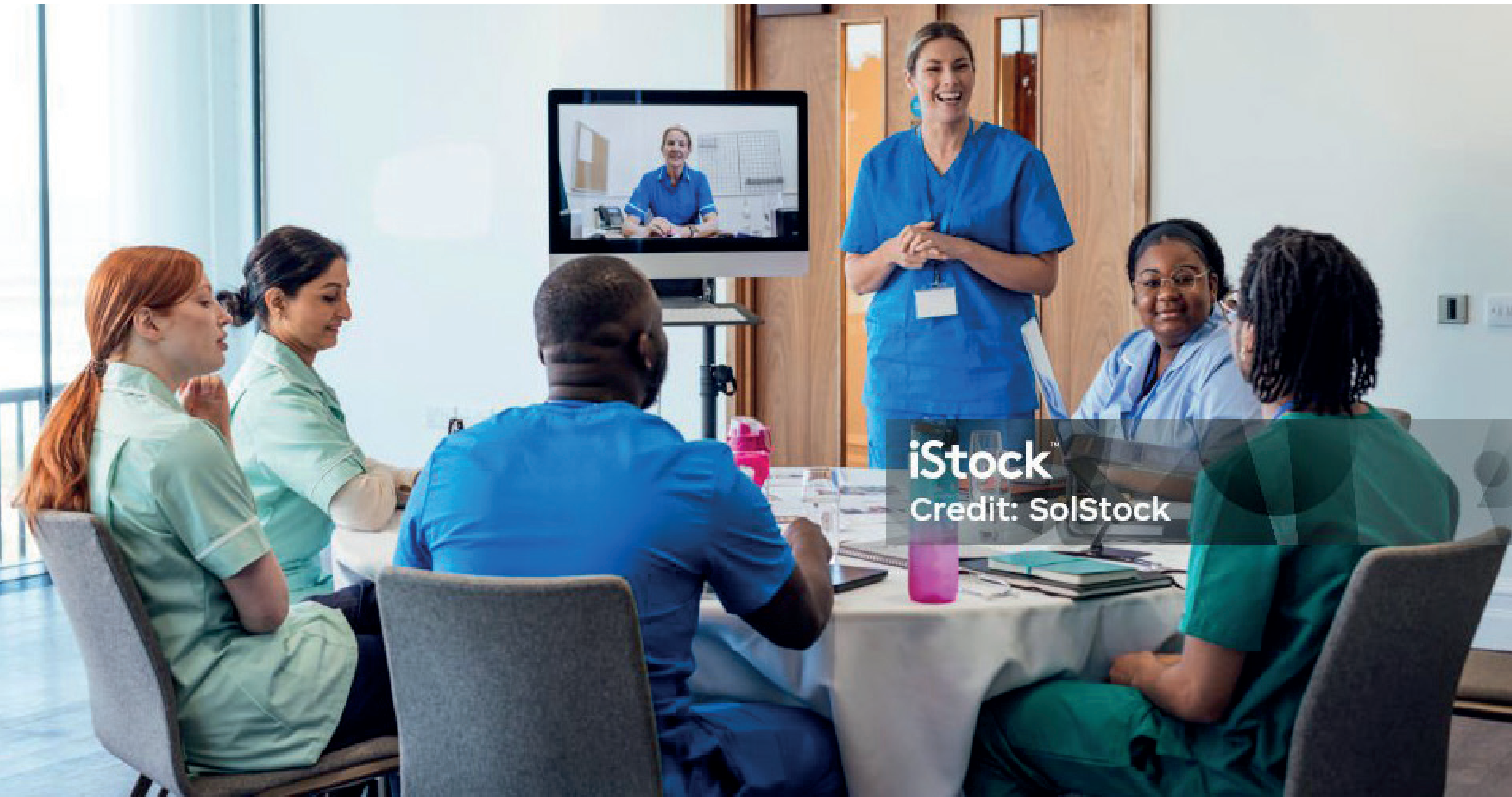

What will be the intervention?

Integration of dental support in the mental health care setting would be helpful considering the complexity of the barriers people with SMI face in maintaining oral health and accessing dental services.

As the way forward for integrating mental and dental health care services, one potential system-level intervention is discussed here that will be delivered in close collaboration with mental and dental professionals, who will get appropriate training to deliver the intervention.

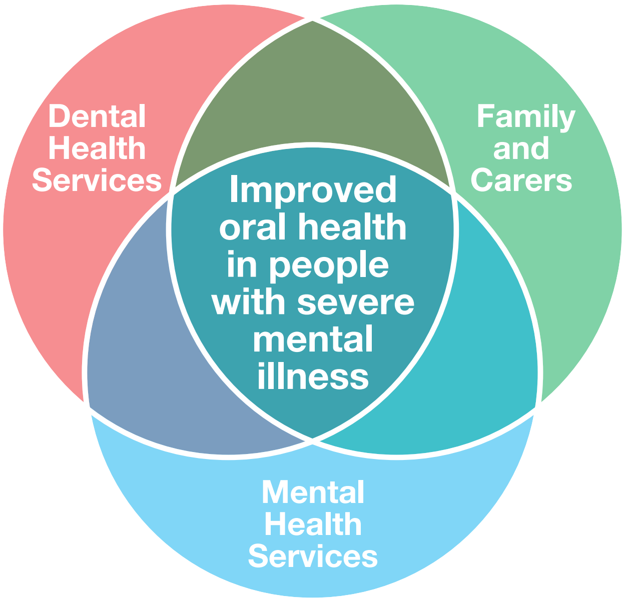

The intervention will be delivered in the mental health care setting. The intervention will include:

1. Dental professionals (preferably dental therapists) visiting mental health care services.
2. Mental health care professionals have a conversation with service users with lived experience of severe mental illness about the importance of oral health, ask them about their oral health conditions and dental service use and talk about available oral health support systems in their mental health care service and will introduce them to the dental therapists
3. A brief dental check-up/screening (if the service user agrees) conducted by the dental therapists
4. Tailored advice on maintaining good oral health including tooth brushing demonstration and tailored advice on maintaining oral hygiene
5. Provide encouragement and support in maintaining good oral health
6. Provide support on dental attendance for maintaining routine dental checkups and those who need dental treatment.

Dental Professionals visiting Mental Health Services

Components of the intervention

Initiating conversations
↓
Dental checklist
↓
Brief oral screening
↓
Tailored advice
↓
Toothbrushing demonstration

Support on dental attendance

Those having regular dental visit will be encouraged to maintain routine dental check-ups and seek dental treatment (if needed)

Those who never visited a dentist and don’t need urgent treatment will be supported to find a dentist for maintaining routine dental check-ups and seek dental treatment (if needed)

Those who need urgent dental treatment will be assisted to seek emergency dental care

Proposed Intervention

Step 1

Initiation of conversion around dental health by mental health care professionals:

When people with severe mental illness (SMI) come to mental health care services, a member of the community mental health team will start the conversation about oral health and briefly mention the importance of oral health.

They will ask the service user about:

- **how they perceive their oral health,**
- **how satisfied they are with their oral health,**
- **when they last visited a dentist**

They will tell the service user that they have a dental therapist visiting the mental health clinic who the service user can speak to about their oral health and how to maintain good oral health if they wish and will introduce them to the dental therapists visiting their mental health care service.

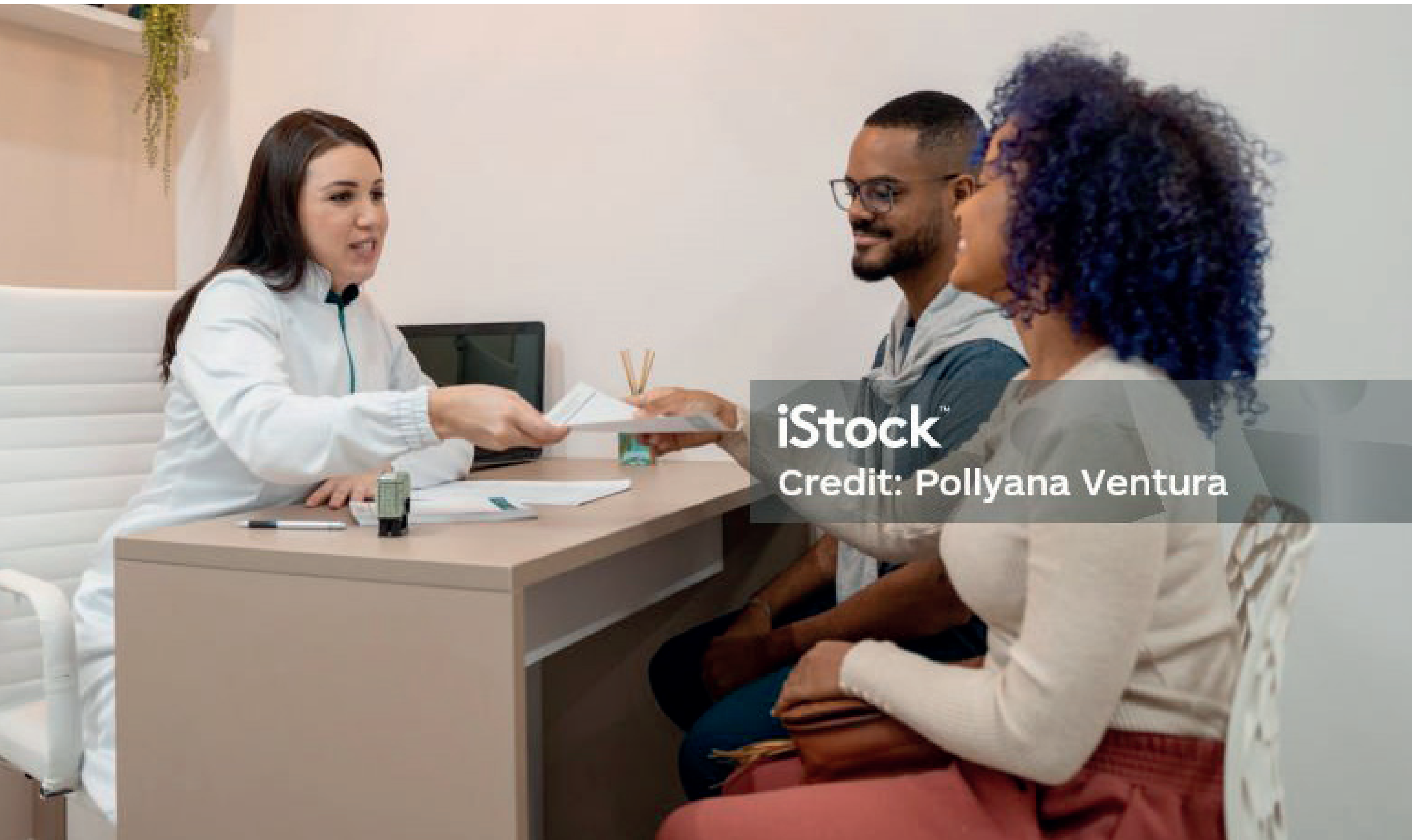

Step 2

As part of the intervention, dental therapists will discuss the person's oral health, oral hygiene practice, and dietary habits regarding sugary food and drink intake.

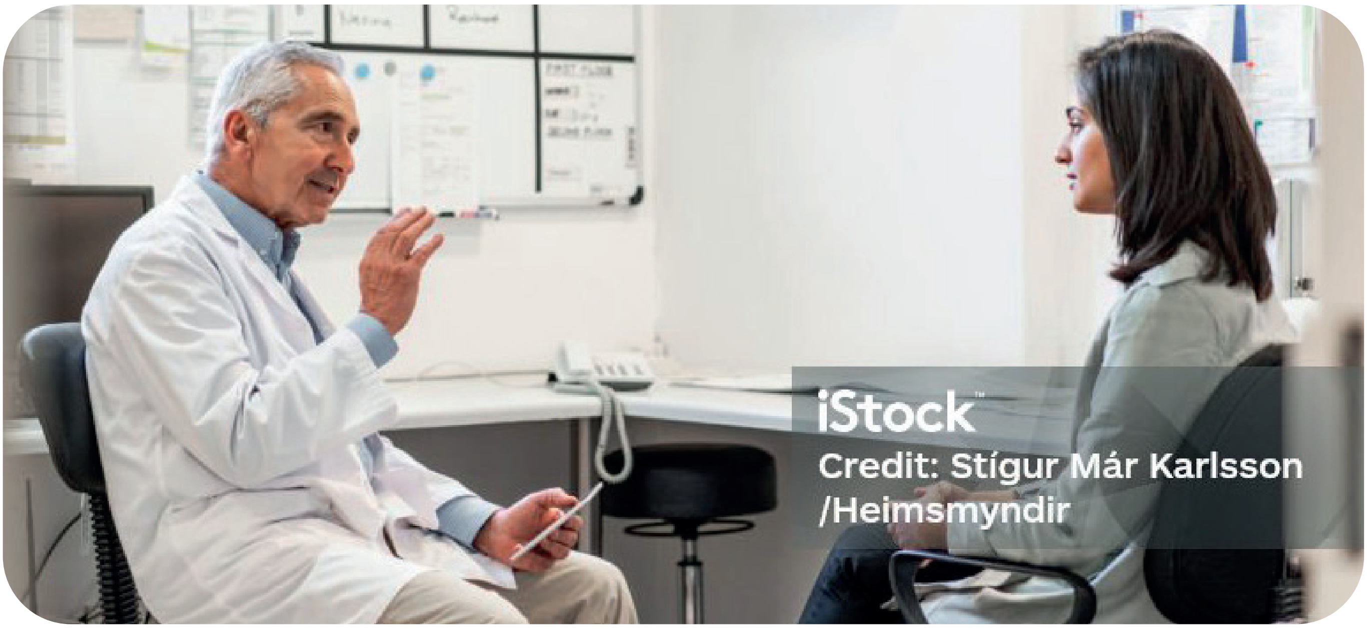

Step 3

The dental therapists will offer to do a brief dental check-up using a dental mirror. If the service user agrees then the dental check-up will be conducted.

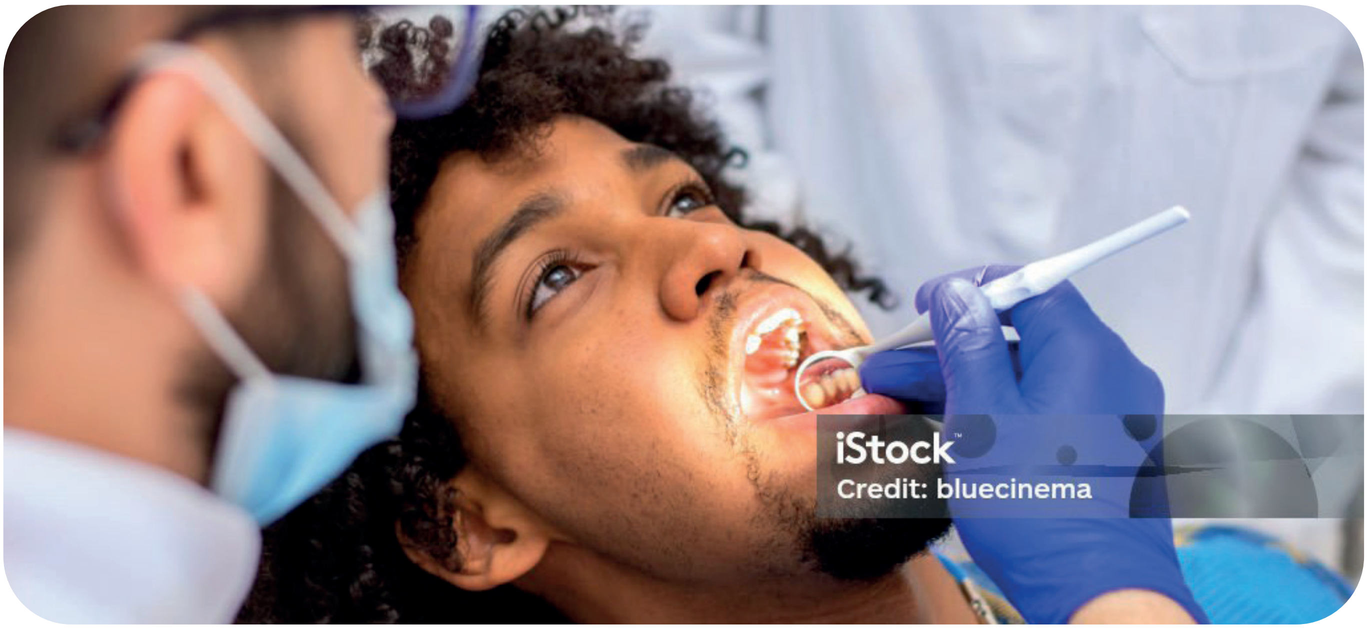

Step 4

The dental therapists will provide tailored good oral health maintenance-related advice, which will include:

1. Importance of regular Tooth brushing. Tooth brushing instruction with details on when and how to brush, along with a practical demonstration tailored to their specific needs.
2. Explain the role of dietary sugar and its relation to oral health and give related tailored advice regarding reducing the frequency and amount of sugary food and drink consumption to maintain good oral health and reduce tooth decay/ dental caries.
3. Explain the role of tobacco and alcohol and their relation to oral health and give tailored advice where needed.
4. Explain how the side effects of some antipsychotic medications are related to oral health, such as dry mouth, and how to deal with it.
5. Explain the importance of routine dental check-up for good oral health.

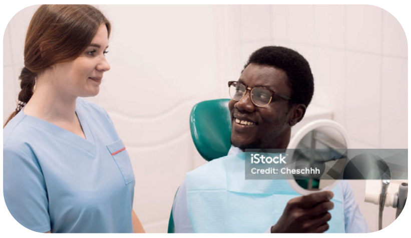

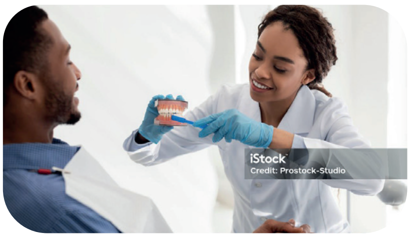

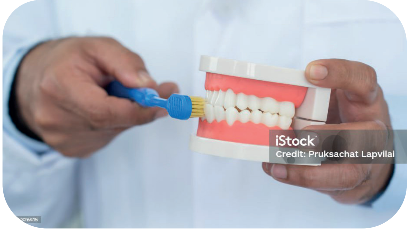

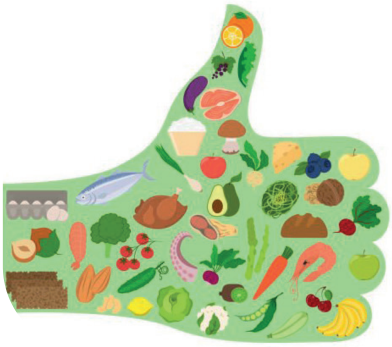

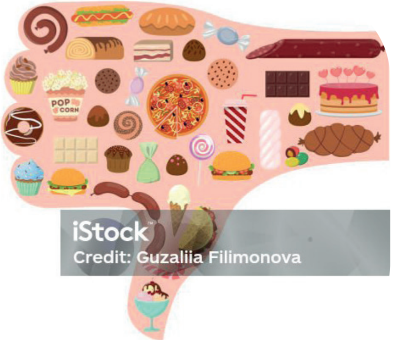

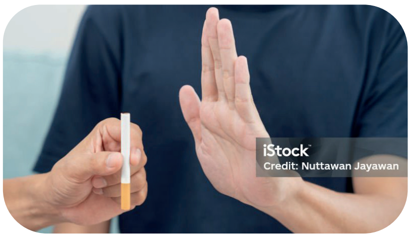

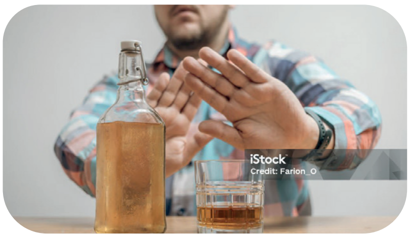

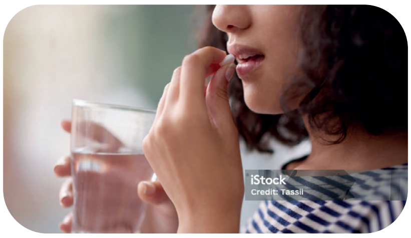

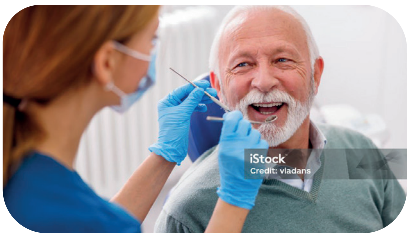

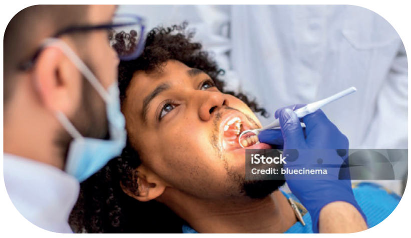

Step 5

For the next four weeks, the service users will receive a weekly phone/text message (as they prefer) with positive reinforcement on regular tooth brushing. Their family members will be involved in the intervention, if the service users wish, to encourage and remind them to maintain regular oral hygiene and accompany them to visit a dentist.

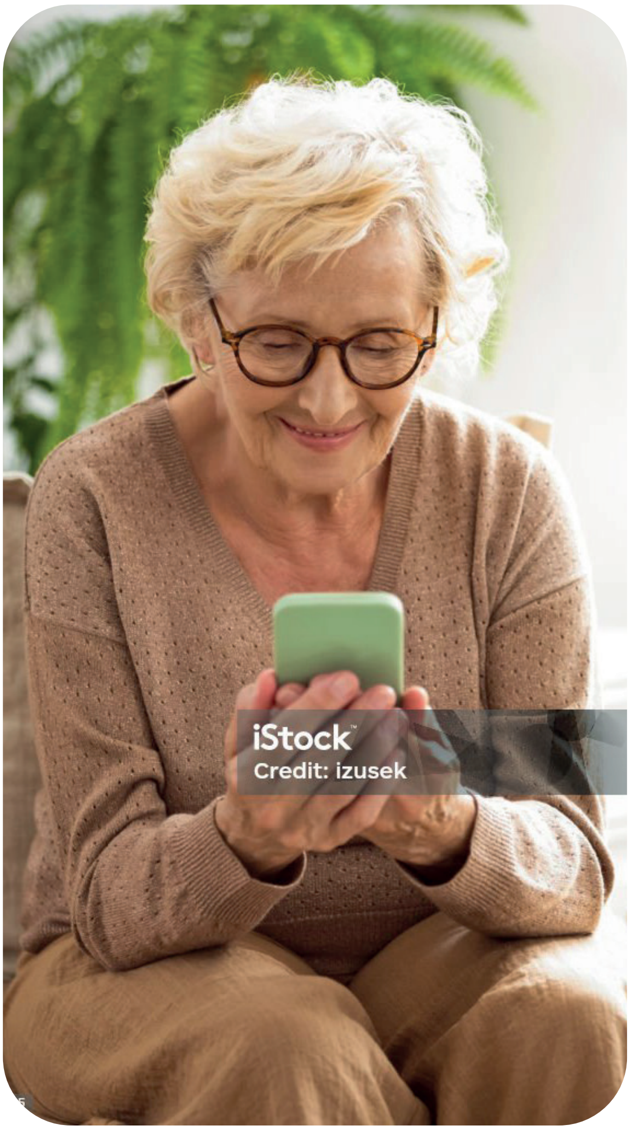

Step 6

Oral health care will be included in their care plan. Members of the community mental health team, with support from the dental therapists, will help facilitate access to a dentist or community dental services.

- Those having regular dental visit will be encouraged to maintain routine dental checkups and seek dental treatment (if needed)
- Those who never visited a dentist and don't need urgent treatment will be supported to find a dentist for doing and maintaining routine dental checkups and seek dental treatment (if needed)
- Those who need urgent dental treatment will be assisted to seek emergency dental care.

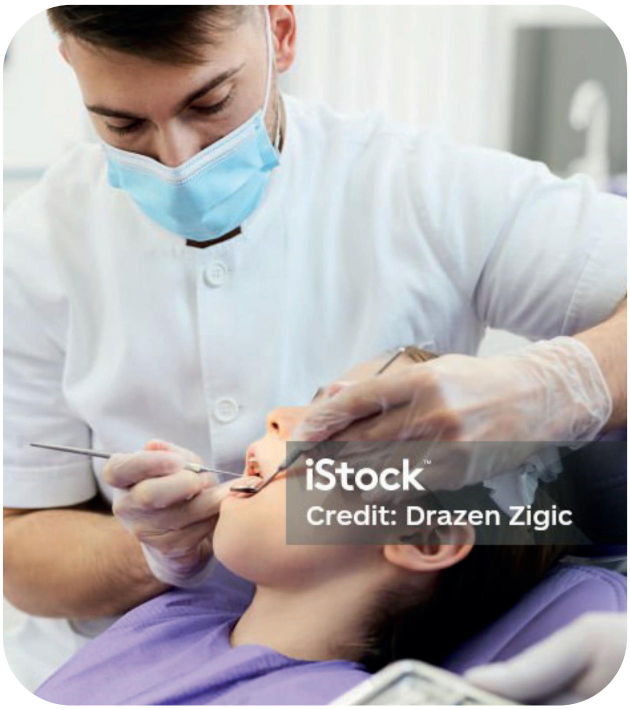

Contact information

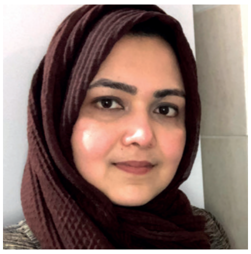

Dr. Masuma Pervin Mishu,
Lecturer in Public Health,
University College London,
Email: Masuma.mishu@ucl.ac.uk
@MPMishu
www.linkedin.com/in/masuma-pervin-mishu-5315813a

More information about the work can be found here: http://tinyurl.com/smileucl

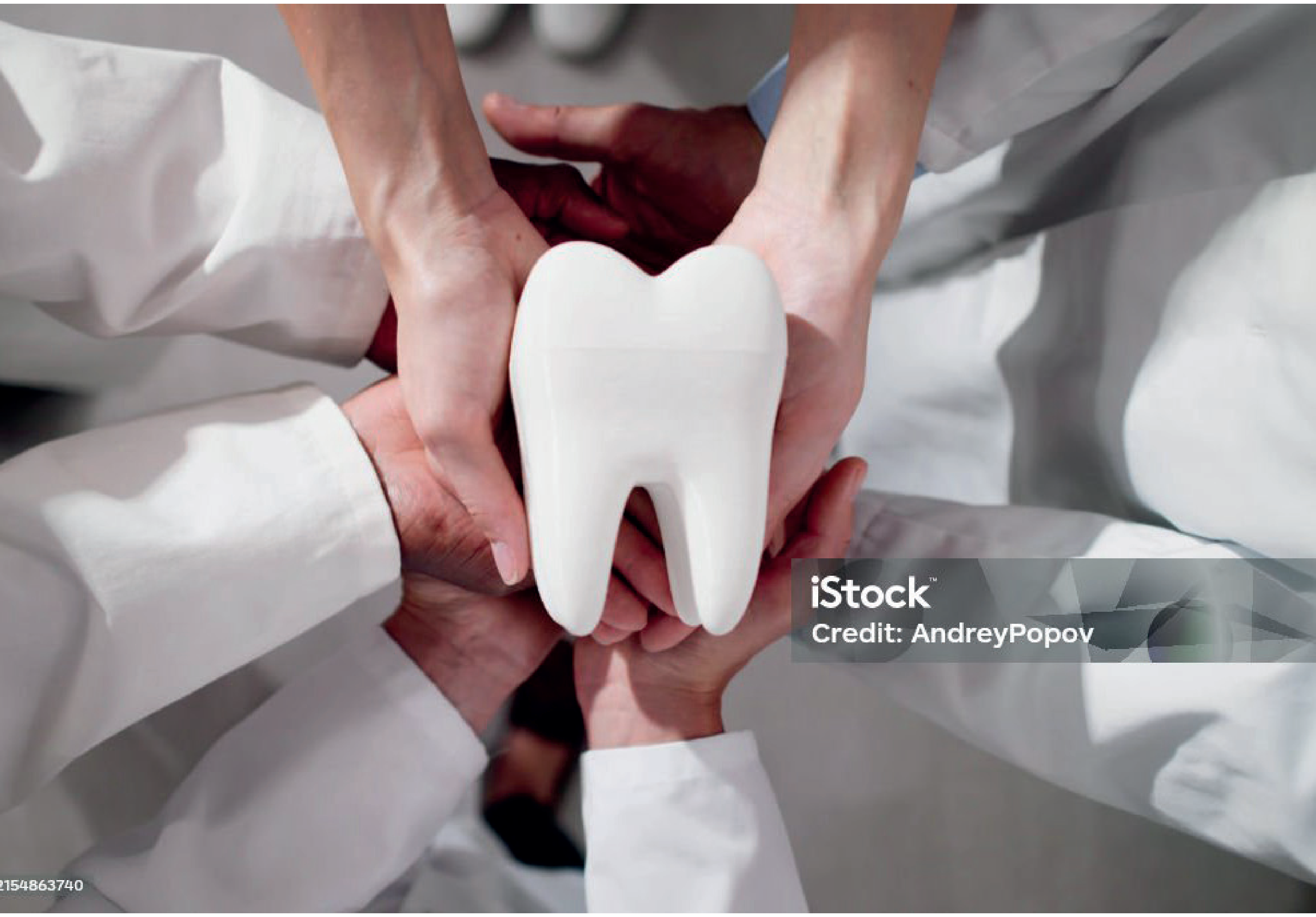

Sheffield Health and Social Care NHS Foundation Trust

Institute of Epidemiology and Health Care
